# Three decades (1978–2008) of Advanced Trauma Life Support (ATLS™) practice revised and evidence revisited

**DOI:** 10.1186/1757-7241-16-19

**Published:** 2008-12-18

**Authors:** Kjetil Søreide

**Affiliations:** 1 Department of Surgery, Stavanger University Hospital, Stavanger, POB 8100, N-4068 Stavanger, Norway

## Abstract

The Advanced Trauma Life Support (ATLS) Program was developed to teach doctors one safe, reliable method to assess and initially manage the trauma patient. The ATLS principles represents an organized approach for evaluation and management of seriously injured patients and offers a foundation of common knowledge for all members of the trauma team. After 3 decades of teaching (1978–2008) of ATLS worldwide one should intuitively perceive that the evidence for the effect of ATLS teaching on the improved management of the injured patient be well established. This editorial addresses aspects of trauma education with needs for further development of better evidence of best practice.

## 

The Advanced Trauma Life Support (ATLS^®^) Program was developed to teach doctors one safe, reliable method to assess and initially manage the trauma patient. The ATLS principles represents an organized approach for evaluation and management of seriously injured patients and offers a foundation of common knowledge for all members of the trauma team The concept is simple, and based on the mnemonic "ABCDE" order of which priority takes place in management of the injured patient: **A**irway and cervical spine protection; **B**reathing; **C**irculation; **D**isability, and; **E**xposure/**E**nvironment. The emphasis is on the critical "first hour" of care, focusing on initial assessment, lifesaving intervention, reevaluation, stabilization, and, when necessary, transfer to a trauma center. Obviously the approach is justified, as about 30% of all inhospital trauma deaths occur within the first hour of injury, and 3 in 4 inhospital trauma deaths occur within the first 48 hours [1].

ATLS was developed by the American College of Surgeons (ACS) following the tragic 1976 event of an orthopedic surgeon piloting his plane, who crashed into a Nebraska cornfield with his family, causing severe injuries to his 3 children and the death of his wife – a story retold by himself 30 years later [2]. Insufficiency in the system was noted by the care received at the primary care facility, leading to a call for a systems change that began in Nebraska [3], and in 1978 the first ATLS course was held [4]. For over three decades (1978–2008) the ATLS course has changed in-hospital management of major trauma patients and is now accepted as a standard of care in over 50 countries worldwide and has been thought to about 1 million physicians, including Europe and Scandinavian countries since the mid 1990s [5-8].

The ATLS^® ^Student Course Manual is updated approximately every four years. The 8^th ^edition was released in October this year, featuring over 100 color images and including a DVD with skills from the course demonstrated in video segments [9]. Practice has been revised according to "best evidence" [9], acknowledging that the principles in ATLS is not necessarily reflecting the forefront of trauma care as practiced in busy, large-volume (academic) trauma centers. Rather, the principles of practice take aim to provide a basic understanding and logic in the safe management of the injured patient independent of institution location and resources. Acknowledging the increasing global impact of ATLS, the review committee has included a broader international panel in the development of evidence-based, expert opinions. Several changes have resulted in the new edition, including a chapter on disaster management, and revisions of recommendations for specific injuries/conditions, such as no current support for the use of steroids in spinal cord trauma and, in pediatric trauma that physiologic changes/blood loss should guide the use of laparotomy/embolization rather than the finding of a splenic injury and a blush on CT per se [9]. For many practicing clinicians dealing with trauma patients these statements will not be new, but nonetheless represents a standard for which the inexperienced or untrained are now taught to manage these conditions.

After 3 decades of teaching, practice and implementation of ATLS worldwide one should intuitively perceive that the evidence for the effect of ATLS teaching on the improved management of the injured patient be well established. Fact is, besides a few studies demonstrating the effect on process of care by mandatory implementation of ATLS training [10-12], and studies investigating the effect of having ATLS skills in a simulation environment [13-15], very little "real-world" evidence exists on the true effect on trauma mortality per se. In a systematic review [16] comparing effectiveness of hospitals with an ATLS-trained trauma response system versus hospitals without such a response system in reducing mortality and morbidity following trauma, the authors found no clear evidence that ATLS training (or similar) impacts on the outcome for victims of trauma. However, there is some evidence that educational initiatives improve knowledge of what to do in emergency situations [16]. Further, there is no evidence that trauma management systems incorporating ATLS training impact positively on outcome [16]. Future research should concentrate on the evaluation of trauma systems incorporating ATLS, both within hospitals and at the health system level, by using rigorous research designs.

Similarly, a systematic review of ATLS in the prehospital setting could find no hard evidence of either positive, nor negative effect on outcome [17] – however, no level I studies were found on the subject, thus hampering drawing any firm conclusions. Further, conclusions may differ according to geographic region and type of crew investigated (ambulance crew vs physicians), e.g. with no difference demonstrated in a large Canadian study on prehospital advanced life support [18], while a positive impact of applying physician-performed prehospital advanced life support in a Norwegian system [19]. Core topics of controversy include perceived high-risk procedures (such as prehospital intubation) which mandates proper training and utility [20].

Obviously, education of advanced trauma life support principles, with the ATLS™ Course in a current leading forefront, has changed how the trained physician thinks and perceives initial evaluation of the traumatized patient, and has been met by enthusiasm in most instances. However, recognition of perceived shortcomings such as the utility and recommendations for diagnostic imaging [21], high costs, low compliance (even among general surgeons in the US) [22], and critique of the predomination of "North-American principles" and the organization's rigidity on change has spurred discussion on the value of ATLS, in particular outside the US [23-26] and even the development of a European alternative course [27]. In addition, supplementary education, including team-training using crew resource management (CRM) principles has been recognized and introduced in Norway [28], and is now implemented alongside ATLS training in a national scale [29]. Alternative training models are made mandatory in areas where high-risk, low-volume life-saving procedures might be performed [30,31]. Just as trauma does not respect the borders of organ systems or medical disciplines, training for the complex management of injured patients needs several approaches and solutions to the educational challenge.

## Competing interests

The author declares that they have no competing interests.

## Authors' contributions

KS perceived the concept and drafted the article.
